# Immunotherapy in Breast Cancer: When, How, and What Challenges?

**DOI:** 10.3390/biomedicines9111687

**Published:** 2021-11-14

**Authors:** Beatriz Henriques, Fernando Mendes, Diana Martins

**Affiliations:** 1 Politécnico de Coimbra, ESTeSC, UCPCBL, 3046-854 Coimbra, Portugal; maria.henriques@estescoimbra.pt (B.H.); fjmendes@estescoimbra.pt (F.M.); 2 Laboratório de Investigação em Ciências Aplicadas à Saúde (LabinSaúde), Politécnico de Coimbra, ESTESC, 3046-854 Coimbra, Portugal; 3 Biophysics Institute of Faculty of Medicine, Coimbra Institute for Clinical and Biomedical Research (iCBR) Area of Environment Genetics and Oncobiology (CIMAGO), University of Coimbra, 3004-504 Coimbra, Portugal; 4 Center for Innovative Biomedicine and Biotechnology (CIBB), University of Coimbra, 3004-504 Coimbra, Portugal; 5 Clinical Academic Center of Coimbra (CACC), 3004-504 Coimbra, Portugal; 6 European Association for Professions in Biomedical Sciences, B-1000 Brussels, Belgium

**Keywords:** breast cancer, immunotherapy, therapeutic resistance

## Abstract

Breast Cancer (BC) is the second most frequent cause of cancer death among women worldwide and, although there have been significant advances in BC therapies, a significant percentage of patients develop metastasis and disease recurrence. Since BC was demonstrated to be an immunogenic tumor, immunotherapy has broken through as a significant therapy strategy against BC. Over the years, immunotherapy has improved the survival rate of HER2+ BC patients due to the approval of some monoclonal antibodies (mAbs) such as Trastuzumab, Pertuzumab and, recently, Margetuximab, along with the antibody-drug conjugates (ADC) Trastuzumab-Emtansine (T-DM1) and Trastuzumab Deruxtecan. Immune checkpoint inhibitors (ICI) showed promising efficacy in triple-negative breast cancer (TNBC) treatment, namely Atezolizumab and Pembrolizumab. Despite the success of immunotherapy, some patients do not respond to immunotherapy or those who respond to the treatment relapse or progress. The main causes of these adverse events are the complex, intrinsic or extrinsic resistance mechanisms. In this review, we address the different immunotherapy approaches approved for BC and some of the mechanisms responsible for resistance to immunotherapy.

## 1. Introduction

Breast cancer (BC) is the second leading cause of cancer-related deaths among women worldwide and one in three women have a risk of developing BC in their lifetime, making BC the leading cause of cancer in the world [1,2]. By 2050, female BC is expected to reach 3.2 million new cases worldwide [3].

Human breast carcinomas are heterogeneous diseases composed of distinct subtypes with different behavior and responsiveness to treatment [4]. The expression of specific immunohistochemical biomarkers, such as hormone receptors (HR) and human epidermal growth receptor 2 (HER2), allows the diagnosis of BC into specific molecular subtypes: Luminal A, Luminal B, HER2-enriched and Triple Negative Breast Cancer (TNBC) type, with distinct phenotype, treatment strategies and clinical results [3,5,6].

Luminal breast carcinomas represent the majority (60–70%) of all BC and are associated with the expression of hormone receptors: estrogen receptors (ER) and/or progesterone receptors (PR) [7]. These carcinomas can be subdivided between the luminal A (HR+/HER2−/low Ki-67 expression) and luminal B (HR+/HER2+/high Ki-67 expression), with the first being less aggressive than the other subtypes [2,6]. Endocrine therapy remains the main therapeutic strategy, and it includes selective ER modulators (SERM), aromatase inhibitors (AI) and selective ER down-regulators (SERD) [8,9].

HER2+ BC is an aggressive and fast-growing subtype representing 20% of BC cases [3,7]. These tumors are characterized by the *HER2/ERBB2* oncogene amplification/overexpression, which causes a higher expression of HER2 and is associated with more invasiveness and recurrence [10,11]. The therapeutic strategy comprises HER2 receptor-targeting medicines, which include anti-HER2 monoclonal antibodies Trastuzumab, Pertuzumab and Trastuzumab–Emtansine, and the dual tyrosine kinase inhibitor Lapatinib [2,6]. The standard systemic therapy for this type of BC is anti-HER2 drugs plus chemotherapy [12].

Breast carcinomas that do not express ER, PR and HER2 are classified as basal or triple-negative breast cancer (TNBC) [1]. This subtype accounts for approximately 10–20% of all BC and represents a high-risk group associated with increased rates of relapse, recurrence and mortality [7,13,14]. The main cause of its increased aggressiveness and the worse clinical outcomes with TNBC is associated with a highly heterogeneous tumor, which does not have a specific therapeutic target [13,14]. Neoadjuvant chemotherapy (NACT) is the standard treatment for patients with TNBC BC [13].

Recently, significant advances were achieved in early detection and therapy in BC, resulting in a 38% decrease in the BC mortality rate [15]. Despite the increase of diagnostics and therapeutic innovation, the success of BC therapy has been a major challenge due to its resistance to treatment. The therapy resistance associated with tumor heterogeneity is the main cause for tumor recurrence and metastasis [8,16,17,18,19]. Roughly 20% of patients with BC will have recurrence or metastasis during the first 5 years [20]. To improve this outcome, it is necessary to explore new therapeutic approaches that offer more effective treatments and prolong the survival of patients [6]. Therefore, immunotherapy has become a new approach for BC, once its main goal is to restore anti-tumor immunity. [11,21,22,23]. Indeed, accumulating data now support a key role for the immune system in determining both responses to standard therapy and long-term survival in patients with BC [1,6,14].

With this review, we aim to discuss the relationship of the immune system with BC and the role of immunotherapy in BC treatment. In parallel, we also address the challenges associated with different resistance mechanisms related to this treatment.

## 2. Breast Cancer Microenvironment

The BC cells are surrounded by different stromal components that have an important role in the BC’s development, in its metastatic ability and its response to therapy [24]. A tumor is much more than clusters of transformed cells standing alone, and the epithelial tumor cells can only develop in an aberrant microenvironment composed of altered extracellular matrix and several non-transformed cells, such as cancer-associated fibroblasts (CAF), adipocytes, endothelial cells, extracellular matrix components, blood vessels and immune cells, all of which compose the tumor microenvironment (TME) [16,24,25].

TME stromal components have different functions and interactions in BC, wherein tumor development can influence the microenvironment, which provide an important support for BC development [16,25,26]. The stromal constituent has an abundance of inflammatory cells and activated fibroblasts expressing extracellular matrix (ECM) components, as well as growth factors that promote the survival and proliferation of tumor cells [27]. In fact, the presence of tumor-infiltrating lymphocyte (TIL) in BC is significantly associated with higher expression of Ki-67, suggesting that the immune response has an important role in tumor progression [28].

The stromal TME cells also secrete a range of chemokines, cytokines and growth factors that can promote different mechanisms, such as proliferation, angiogenesis, inhibition of apoptosis, immune system suppression and evasion of immune surveillance. These mechanisms will influence the behavior of tumor cells and contribute to the growth, progression and metastasis of BC [16,25,26]. These capabilities of tumor cells allow the TME to be dynamic and constantly adapt to new microenvironment conditions [24,26]. For example, Cancer Associated Fibroblasts (CAFS) are associated with tumor formation and metastasis in human breast cancers [27]. They are essential for ECM deposition and remodeling. through the synthesis of several ECM components and ECM degradation proteases such as matrix metalloproteinases (MMPs) [27]. Besides CAFs, myoepithelial cells are also a key component of the stromal tumor microenvironment. The inhibition of tumor growth and invasion of BC cells is associated with paracrine factors secreted by myoepithelial cells [27]. These factors include ECM proteins, protease inhibitors and several growth factors [29].

Myoepithelial cells are also associated with the expression of extracellular matrix structural proteins and they accumulate extracellular matrices [27].

Stroma has also been correlated with clinical outcomes and response to therapy in BC [27]. The expression of ECM genes may classify BC into different subgroups [30]. Moreover, single-cell analysis identified the phenotypes of tumor and stromal single cells and revealed multicellular features of the tumor microenvironment associated with distinct clinical outcomes [31].

## 3. Immune System and Breast Cancer

The immune system plays an important role in normal breast development, where diverse populations of immune cells are present in the breast stroma at various stages of development and maturation, like post-natality, puberty and pregnancy [6,32,33,34].

BC is usually classified as a moderately immunogenic cancer, with the HER2+ and TNBC subtypes considered to be the most immunogenic subtypes [11,22]. Immune cells have a crucial role in BC recognition and early eradication, but also in tumor progression [11,35]. The process that describes the interactions between the host’s immune system and the tumor’s cells is denominated as immunoediting, and it englobes three phases: the “elimination”, the “equilibrium” and the “escape” [11,15]. Mendes et al. proposed a fourth phase, the “exhaustion” [36].

Immunoediting commences by recognizing cancer cells as foreign and mounting an immune response against them—the elimination phase [11,15,36]. This starts with breast tumorigenesis, in which the tumor tissue releases signals that activate the innate immunity cells such as Natural Killer (NK) cells, macrophages and neutrophils, all acting to eliminate the tumor cells. This process also contributes to dendritic cell (DC) maturation, that are antigen-presenting cells (APC) to T cells and that initiate a specific immune response (adaptative immunity) against the tumor, mainly with the CD8+ cytotoxic T cells (CTL) [11,15,22,37].

Some BC tumor cells variants (less immunogenic) can escape from the elimination phase and remain dormant while immune cells prevent tumor cell growth, reaching the “equilibrium” phase. Another proposed phase is exhaustion, where immune cells may become dysfunctional and remain in a state of T cell “exhaustion”. Retaining a high potential for a successful defence against cancer, this indicates that several T cells are not entirely and permanently exhausted but can be mobilized to become highly functional [36]. However, the BC can also “escape” from the immune recognition and elimination and/or exhaustion phases through different mechanisms, and continue to aggressively proliferate [11].

### Mechanisms of Immune System Evasions in Breast Cancer

The tumors cells have the capability to evade the immune system through the development of different mechanisms. A less efficiently surface antigen expression and a reduced expression of the major histocompatibility complex (MHC) in tumor cells, which can decrease immune detection and immune cell activation, contributes to the evolution a clone selection of low immunogenic tumor cells that escape immune system surveillance [11,14,38]. Other mechanisms to escape immune detection are associated with changes to an immunosuppressive TME induced by regulatory T cells (Treg), myeloid-derived suppressor cells (MDSC) and tumor-associated macrophages (TAM). These mechanisms also include the upregulation of immune checkpoint receptors, such as cytotoxic T-lymphocyte-associated protein-4 (CTLA-4), programmed cell death protein-1 (PD-1), programmed cell death protein-Ligand 1 (PD-L1) and T-cell immunoglobulin 3 (Tim-3) and the presence of tumor-derived immunosuppressive factors [11,14,22,38,39]. The previous mechanisms are probably caused by the combination of genetic instability inherent in all tumor cells along with the immunoselection process [40].

## 4. Immunotherapy in Breast Cancer

Although BC is not considered one of the most immunogenic cancers, like melanoma and lung cancer, immunotherapy is a highly emerging form of BC therapy [14,23].

Immunotherapy is not a new approach to BC therapeutic strategies, as some monoclonals antibodies (mAb) have long been used as therapeutic agents to treat BC tumors, with a passive immunotherapy like Trastuzumab as an example. In recent years some other modalities, like antibody-drug conjugates (ADC) and immune checkpoint inhibitors (ICI), have increasingly been studied as a preventative approach and treatment for BC [11].

### 4.1. Monoclonal Antibodies

Humanized mAb were the first immunotherapy treatments to be developed and have been enhanced as an immunologically based therapeutic strategy. They are IgG isotypes that bind and neutralize a target molecule expressed by cancer cells, which their survival and proliferation rely on [41,42].

mAb have been developed against a great variety of targets, such as the HER2 pathway, ICI, human epidermal growth factor receptor (EGFR), vascular endothelial growth factor (VEGF) and others, and some of these are approved to be used as a therapy for BC while others continue under study [43].

**Figure 1:**
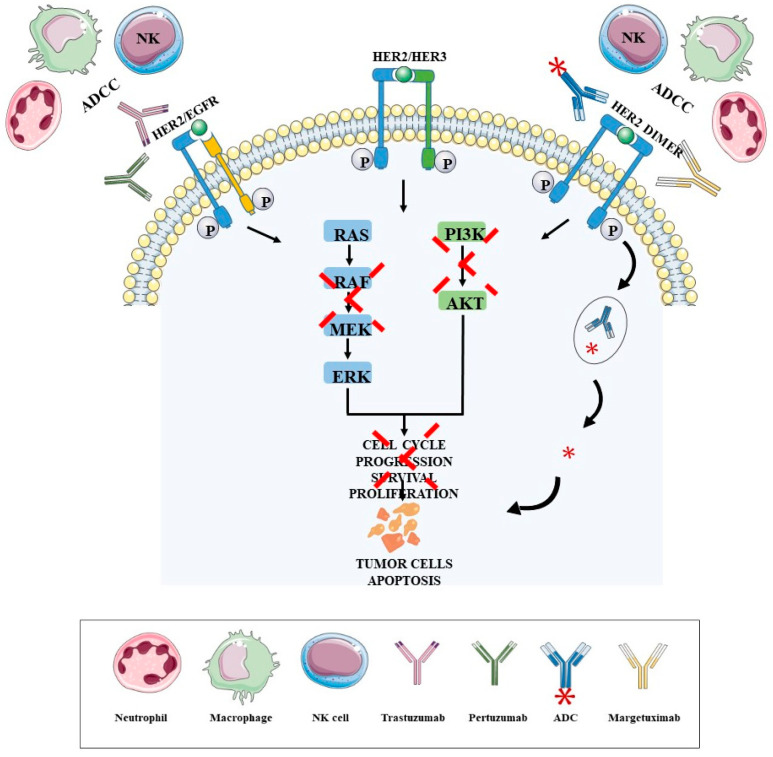
Immunotherapy modalities approved as a treatment for patients with HER2 BC and their mechanisms of action. Trastuzumab, Pertuzumab and Margetuximab are the mAbs approved for HER2 BC therapy directed for HER2 protein in tumor cells, specifically for the domain II (Pertuzumab) and domain IV (Trastuzumab). When the mAbs attach, the HER2 protein blocks the dimerization of HER2 protein with the other receptors (HER2, HER3 or EGFR), inhibiting the activation of the HER2 protein and consequently the activation of the PI3K and MAPK pathways, which leads to an increase in cell cycle arrest, the suppression of cell growth and proliferation, causing the apoptosis of tumor cells. These mAbs can also activate both innate and adaptive immune systems, inducing the ADCC that allows to kill HER2-overexpressing cells via NK cells, macrophages and neutrophils, and eliciting an adaptive immune response based on HER2 presentation by MHC-I molecules to activate the anti-tumor activity of CTL. Other immunotherapies approved are the Trastuzumab-Emtansine and Trastuzumab Deruxtecan, that are an ADC who consists in a therapy that conjugates the Trastuzimab covalently conjugated with the cytotoxins DM1 (Trastuzumab-Emtansine), a microtubule polymerization inhibitor, and topoisomerase I inhibitor payload (Dxd) (Trastuzumab Deruxtecan), responsible for the inhibition of DNA replication. The Trastuzumab directs the ADC to the tumor cells that express HER2 protein and when the Trastuzumab binds to the HER2 protein the ADC goes into the cell by receptor-mediated endocytosis and the lysosomes degrade the Trastuzumab, realizing the DM1 or the Dxd. The DM1 inhibits the microtubule function and the Dxd inhibits the topoisomerase I, causing the cell cycle arrest and tumor cell apoptosis. Abbreviations are as follows: Antibody Dependent Cytotoxic Cell (ADCC), Antibody Drug Conjugate (ADC), Natural Killer (NK), Human Epidermal growth Receptor 2 (HER2), Human Epidermal Growth Receptor 3 (HER3), Human Epidermal Growth Factor Receptor (EGFR), Phosphatidylinositol-3-kinase (PI3K), Extracellular regulated kinases (ERK), Mitogen-activated protein kinase kinase (MEK), Protein kinase B (AKT).

One of the firsts mAbs to be humanized was Trastuzumab—an IgG1 mAb—that has a direct target of the domain IV of HER2 protein (Figure 1) in BC [41,43]. It has been approved for metastatic HER2+ BC in combination with chemotherapy and has become the conventional therapy for patients with HER2+ early and late-stage BC [11,41,44,45]. Trastuzumab has the capacity to block HER2 signalling through induction G_1_ cell-cycle arrest and inhibition of phosphatidylinositol-3-kinase (PI3K) pathway, which induces apoptosis and angiogenesis inhibition. These mAb can also activate both innate and adaptive immune systems, inducing antibody-dependent cytotoxic cellular (ADCC) killing of HER2-overexpressing cells via NK cells; they can also elicit an adaptive immune response based on HER2 presentation by MHC-I molecules (Figure 1) to activate the anti-tumor activity of CTL and reduce Treg cells in tumor [22].

Another mAb that have been developed and approved against HER2+ BC, is Pertuzumab, in combination with Trastuzumab and conventional treatments [22,41,43,46,47,48]. Pertuzumab is also an IgG1 mAb directed for the domain II of HER2 protein, preventing HER2 heterodimerisation with human epidermal growth receptor 3 (HER3) and EGFR, that impact the intracellular HER2 signalling, and can elicit ADCC [46,49].

Recently, Margetuximab, a new generation mAb, has been approved in combination with chemotherapy for the treatment of patients with metastatic HER2+ BC, with results obtained through SOPHIA clinical trial (Table 1) [22,50,51,52,53]. Margetuximab is a chimeric IgG1 mAb targeting the HER2 pathway and, in preclinical studies, demonstrated an increased capacity to mediate ADCC executed by effector cells such as NK cells, macrophages and neutrophils, compared with Trastuzumab [53].

### 4.2. Antibody-Drug Conjugate

Another novel approach is the antibody-drug conjugate (ADC), which consists of tumor-specific mAb covalently conjugated with a cytotoxin, that act in the microtubules and that have the ability to minimizes drug toxicity and augment antitumor immunity [7].

Trastuzumab-Emtansine (T-DM1) is an ADC constituted by Trastuzumab, that is directed for cells that express the HER2, and the cytotoxic component DM1, a microtubule polymerization inhibitor [11]. T-DM1 has been approved by European Medicines Agency (EMA) and FDA as a single treatment agent for patients with HER2+ metastatic BC progressed and adjuvant treatment of patients with HER2+ early BC who have the residual invasive disease both after therapy with Trastuzumab and chemotherapy [11,14,67]. This therapy has the capacity of inducing the ADCC due to a remarkable chemotherapeutic potency [7,67].

Trastuzumab Deruxtecan is an ADC recently approved by FDA for unresectable or metastatic HER2+ BC and the DESTINY-Breast01 contribute for the approval (Table 1) [68,69]. This ADC consist of an IgG1 mAb, Trastuzumab, covalently conjugated with topoisomerase I inhibitor payload (DXd), and is responsible for the inhibition of DNA replication, cell cycle arrest and inducing tumor cell apoptosis [68].

Recently another ADC was approved for TNBC BC, namely Sacituzumab Govitecan-hziy, due to IMMU-132-01 clinical trial results, as observed in Table 1 [55,70]. This ADC is formed by the conjugation of SN-38, a topoisomerase I inhibitor, with the humanized monoclonal antibody hRS7 IgG1 directed for antitrophoblast cell-surface antigen 2 (Trop-2), and has demonstrated the promotion of ADCC [55,71]. Trop-2 is overexpressed in approximately 90% of TNBC tumors and correlates with poor prognosis in BC due to the induction of pro-oncogenic signalling [55].

ADC therapy is under development and study, targeting different markers expressed in HER2+ BC and TNBC. Most of the agents in development target HER2, like Trastuzumab Duocarmazine, MM-302 and RC48-ADC, but some include targets present in TNBC, like Ladiratuzumab Vedotin and Cofetuzumab.

### 4.3. Immune Checkpoint-Inhibitors

Immune checkpoints are one of the intrinsic controls of immunity, induction anergy or apoptosis of immune cells to maintain immune response homeostasis [23,37,49].

In some tumors, it is observed that the suppression of anti-tumor T cell activity, inhibiting the response against the tumor through the interaction between T cell receptors and co-receptors present in the cancer cell such as immune checkpoints, allows the escape from host immune surveillance [6,14,23].

**Figure 2:**
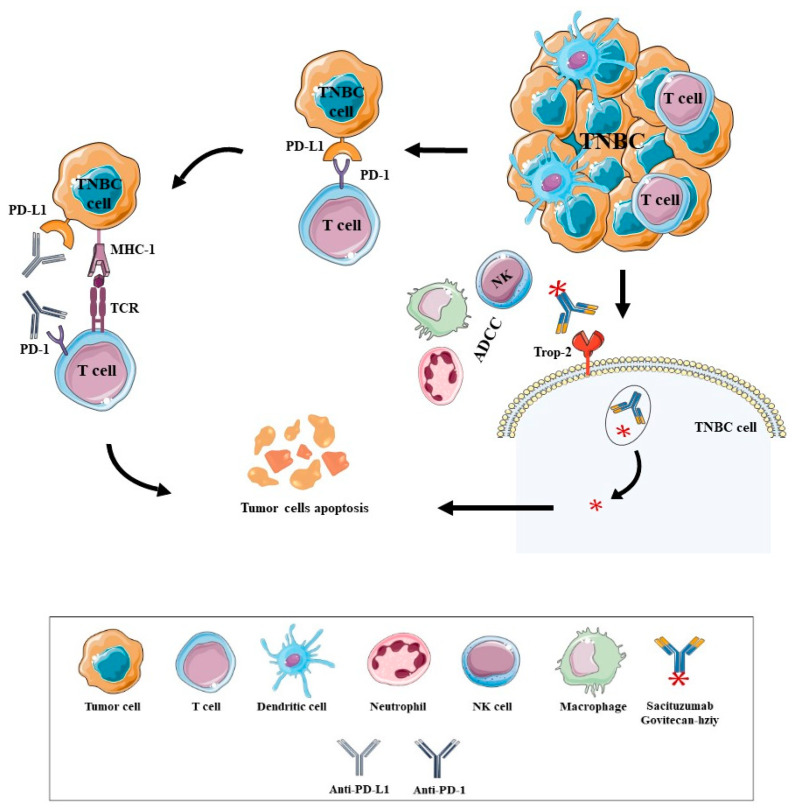
Immunotherapy modalities approved as a treatment for patients with TNBC and their mechanism of action. The tumor cells can escape from host immune surveillance by the expression of immune checkpoint proteins, like PD-L1, that when bound to PD-1 expressed in active T cells, cause the decrease of T cell proliferation and survival. One of the strategies to restore antitumor immune responses and promote immune-mediated elimination of cancer cells are the ICI. Nowadays, Pembrolizumab (anti-PD-1) and Atezolizumab (anti-PD-L1) are two ICI approved for TNBC patients that when bound to their target blocks the interaction of PD-1 and PD-L1 and allows the CTL to eliminate the tumors cells. The Sacituzumab Govitecan-hziy was also approved as a treatment for TNBC patients, an ADC formed by the mAb hRS7 directed for antitrophoblast cell-surface antigen 2 (Trop-2), that was expressed in 90% of TNBC tumors, conjugated with SN-38, a topoisomerase I inhibitor. The hRS7 binds to the Trop-2 expressed in TNBC cells and the ADC goes into the cell by receptor-mediated endocytosis. The lysosomes degrade the mAb, realizing the SN-38 and inhibiting the topoisomerase I, causing the cell cycle arrest and tumor cell apoptosis. It was also demonstrated that Sacituzumab Govitecan-hziy promotes ADCC that kills the tumor cells that express the Trop-2. Abbreviations are as follows: Triple-Negative Breast Cancer (TNBC), Programmed Cell Death Protein-1 (PD-1), Programmed Cell Death Protein Ligand 1 (PD-L1), Major Histocompability Complex 1 (MHC-1), T cell Receptor (TCR), Antitrophoblast Cell-Surface Antigen 2 (Trop-2), Antibody Dependent Cytotoxic Cell (ADCC), Natural Killer (NK), Antibody Programmed Cell Death Protein-1 (Anti-PD-1), Antibody Programmed Cell Death Protein Ligand 1 (Anti-PD-L1).

PD-1 is one of these receptors that can be expressed in active T cells, and when PD-1 binds to PD-L1 (Figure 2) that can be expressed in tumor cells, it causes the decrease of T cell proliferation and survival, cytokine production and other effector functions [41,49].

Another of these receptors that can be expressed and activated on T cells CTLA-4, when it is necessary to limit T cells activation through the competition with the homologue CD28 for ligands CD80 and CD86 expressed in APC, during the primary phase of the immune response [15,41]. The high expression of CTLA-4 in BC cells allows the tumor to suppress the maturation and function of DC and, consequently, decreases T cells function [49].

As such, one of the strategies to restore antitumor immune responses and promote immune-mediated elimination of cancer cells are the ICI [14,41]. Pembrolizumab and Nivolumab are ICI developed against PD-1 receptors and IgG4 antibodies that block the interaction of PD-1 and its ligands, preventing immune-cell deactivation and suppression. The ICI that targets the PD-L1, preventing the interaction with PD-1, are Atezolizumab and Durvalumab, IgG1 mAb, along with Avelumab—also an IgG1 mAb—that can induce ADCC. The anti-CTLA-4 Tremelimumab and Ipilimumab, IgG2 and IgG1 mAb respectively, block CTLA-4 interaction with CD80/CD86 on APC, allowing CD28 to bind to CD80/CD86 and activate the T cell, probably enhancing de novo responses [22,41,43,62].

BC has demonstrated in some studies that the HER-2+ BC and TNBC have high levels of PD-L1 expression and that BC also can express high levels of CTLA-4 [13,72]. ICI are being increasingly explored as a potential treatment strategy for BC and many clinical trials have been developed that some show promising results (Table 1) [41]. The IMpassion130 clinical trial contributed to the approval of the atezolizumab with nab-paclitaxel for the treatment of patients with locally advanced or metastatic TNBC, whose tumors are positive for PD-L1 expression (≥1%) as observed in Table 1 [23,41,73]. Moreover, the Food and Drug Administration (FDA) recently approved Pembrolizumab in combination with chemotherapy as a treatment for patients with untreated recurrent inoperable or metastatic TNBC and with untreated early-stage TNBC, due to the KEYNOTE-355 and KEYNOTE-522 trials results (Table 1) [74].

#### Predictive Biomarkers of ICI Response

The expression of certain biomarkers in TME contributes to the prediction of patients who will have a better benefit from ICI therapy, being important to the identification of such biomarkers for survival rate improvement [75]. In recent studies, some biomarkers have been identified, like tumor-infiltrating lymphocytes (TIL) and PD-L1 expression, contributing to the BC immunogenicity and the immune response complexity [22,76].

The TIL consists of all the lymphocytic cell populations, like CD4^+^ helper T cells (Th1), CTL, B cells and NK cells, that have surrounded the tumor tissue, and the presence of these immune cells are considered abundant in TNBC and HER2+ tumor patients [6,11,22,40,77,78]. The elevated levels of TIL and its specific subsets in TME allow the response’s prediction to anticancer immunotherapies, like ICI, and contribute to better outcomes with standard treatment in terms of response to neoadjuvant chemotherapy [6,22,41,77,78,79].

The PD-L1 expression is the main biomarker to predict the benefit for ICI therapy and their expression is generally positively associated with the presence of TIL [39,41,75].

An emerging predictive biomarker is the tumor mutational burden (TMB), which is defined as the total number of coding and somatic mutations accumulated in tumor cells that are responsible for the emergence of the tumor-specific-antigens (neoantigens) [80]. The presentation of these neoantigens in the surface of tumor cells allows the recognition of these cells as a “non-self” by the immune cells and unleashes the immune-mediated attack [75]. As such, tumors with high TMB have a higher probability of exhibiting more tumor neoantigens, making them more immunogenic and, consequently, increasing the number of TIL in TME and enhancing the immune response against the tumor [80,81,82]. Studies of patients with melanoma and lung cancer demonstrated that tumors featuring higher TMB levels have an improvement in the responsiveness to ICI, making TMB a predictor of response to ICI [81].

BC has intermediate levels of mutational load where the mean somatic mutation frequency of BC is ten times lower than the melanoma and lung tumors. However, among BC subtypes there is high variability of TMB, with HER2+ and TNBC subtypes having a higher mutational load [22]. In the study of Mei et al., the BC with higher TMB showed greater amount of TIL, suggesting an association between TMB and the immune reaction [80].

Different factors have been described as responsible for the increase of TMB in tumors, wherein, according to some studies, the main processes associated with hypermutation in BC are the apolipoprotein B mRNA editing catalytic polypeptide-like (APOBEC) activity and DNA repair pathways alterations signatures [80,82,83]. In the study by Barroso-Sousa et al., the patients with hypermutated BC showed objective and durable response following Pembrolizumab therapy, wherein the APOBEC activity signature was the dominant process identified, followed by the deficient mismatch repair (dMMR) signature [82]. Additionally, in the study conducted by Takahashi el al., higher levels of TMB are associated with increased neoantigen load, and most tumors with high levels of TMB presented an elevated expression of APOBEC3B [83]. In a study, the non-small-cell lung tumor patients who presented the APOBEC signatures obtained a durable clinical benefit after immunotherapy [82,84].

Recently, the FDA approved Pembrolizumab to be used in patients with unresectable or metastatic solid tumors with high TMB (≥10 mutations/megabase) that have progressed following prior treatment with no alternative treatment options [75].

## 5. Resistance to Immunotherapy

Until now, some of the different immunotherapy drugs, that have been previously referred, are approved for different types of cancer such as advanced melanoma, NSCLC, renal cell carcinoma, head and neck squamous carcinoma, lymphomas, bladder cancer and BC [85].

Despite increasing the rates of benefit and survival in tumor patients, some patients do not respond to initial immunotherapy (primary resistance) and some responders inevitably relapse or progress after a period of treatment due to the development of resistance to treatment (acquired resistance) [85,86]. A new type of resistance called adaptive resistance is recently under discussion, wherein the tumor gains the ability to adapt to the immune attack by change itself, which can occur as a primary or an acquired resistance [85,86].

The tumor’s immune resistance comprises very complex tumor-cell-intrinsic and tumor-cell-extrinsic mechanisms that involve the combination of metabolism, genes, abnormal neovascularization, inflammation and other aspects [85,86].

### 5.1. Intrinsic Mechanisms Resistance to Immunotherapy

**Figure 3:**
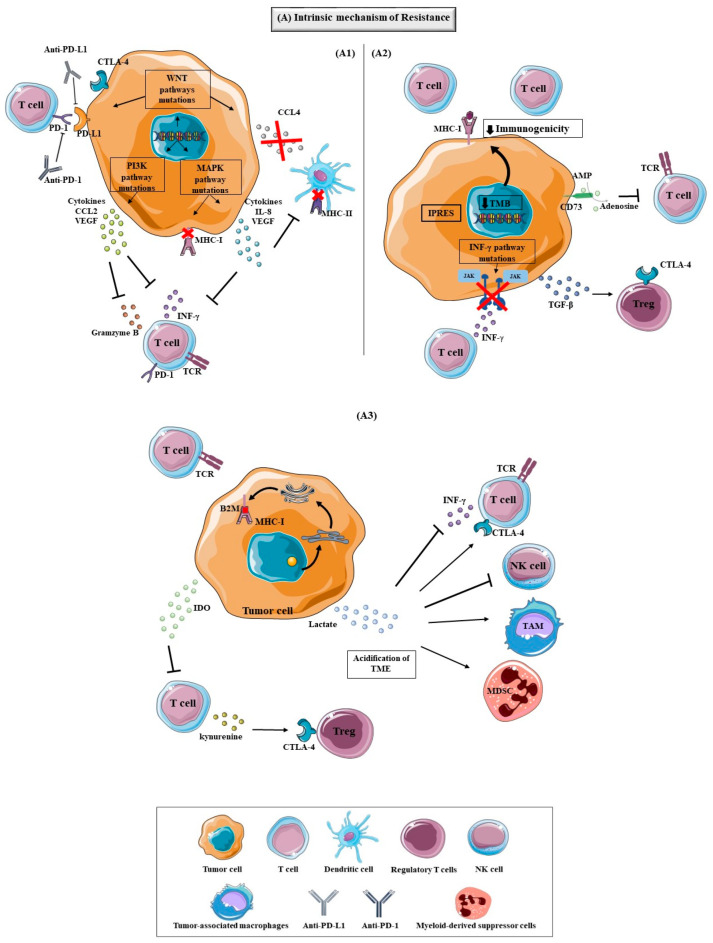
Intrinsic mechanisms of tumor resistance to immunotherapy: (**A1**) The tumor cells have the ability to not respond to immunotherapy by the development of different mechanisms that have been described in different studies. One of the mechanisms that can lead to the resistance are the alterations of some signaling pathways in tumor cells that can cause the reduction or loss of antigens expression, preventing immune cell infiltration or function. The WNT signaling is one of these pathways that can be aberrantly activated in a variety of tumors and act in the tumor cell genesis, proliferation, invasiveness, metastatic and immune microenvironment regulation. In some studies, it was demonstrated that the WNT signaling can control the expression of PD-L1 and CTLA-4 and can reduce the expression of chemokine that attracts CD103+ DC (CCL4), decreasing the DC migration into the TME and contributing to the inhibition of activation and cytotoxic effects of T cells; The PI3K pathway is one of the responsible pathways that contributes to the development and growth of tumors and alterations in their intermediaries, like tumor suppressor protein phosphatase and tensin homolog (PTEN) loss can cause the release of anti-inflammatory cytokines, such as C-C Motif Chemokine Ligand 2 (CCL2) and VEGF that reduce the infiltration of CTL cells in tumors, decrease IFN-γ and granzyme B expression; MAPK signaling is very involved in the tumors development, wherein alterations in this pathway can suppress the expression of MHC-I and MHC-II and the induction of the expression of VEGF and a variety of inhibitory cytokines, such as the interleukin (IL)-8 proteins that inhibit T cell function and recruitment. (**A2**) The mutational load that tumors present can influence the expression of neoantigens and the tumors with low levels of TMB are considered less immunogenic, since they present low expression of neoantigens and a low number of TILs in TME, decreasing the immune response against the tumor. In a study with melanoma patients the expression of a group of transcriptomic signatures, referred as innate anti-PD-1 resistance signature (IPRES), by the tumors can regulate different processes, like mesenchymal transformation, angiogenesis, extracellular matrix remodeling and others, leading the tumor to not respond to anti-PD-1 therapy. The IFN- γ pathway is essential for the immune response, since the IFN- γ secreted by the T cells and APC directed for tumor cells contributes to the immune cell activation and regulation of T cell responses, and can also directly induce tumor cell death. The downregulation or mutations in the molecules involved in the IFN-γ signaling pathway, like JAK1/2 mutations by tumor cells, cause the survival of the tumor cells by resisting the antiproliferative effects of IFN-γ can lead to the tumor’s resistance to the antiproliferative effects of IFN-γ to escape the influence of IFN-γ; Tumor cells can realize immune-suppressive cytokines, like the transforming growth factor (TGF)-β, that can stimulate Treg cells, promoting the angiogenesis and immunosuppression of immune response. The CD73 can form adenosine by the dephosphorylation of adenosine monophosphate (AMP) and the adenosine and CD73 can inhibit the proliferation and function of T cells; (**A3**) the B2M protein is essential to transport MHC class I to the cell surface and mutation in this protein, disrupt the antigen presentation and, consequently, the recognition of CTL fails, which can lead to acquired resistance to immunotherapy. The IDO enzyme liberated by the tumor can reduce the T cell proliferation, inhibiting the activity of T-cell against tumor cells by the degradation of tryptophan into the kynurenine, and kynurenine stimulates the proliferation of Treg cells. The lactate produced by the tumor cells due to metabolic reprogramming can cause the acidification of the TME, affecting the IFN-γ production, NK activation and the amount of MDSC, causing the decrease of the immune response and contributing to the tumor growth. Abbreviations are as follows: T cell Receptor (TCR), Major Histocompability Complex 1 (MHC-I), Major Histocompability Complex 2 (MHC-II), Beta-2-Microglobulin (B2M), Interferon Gamma (IFN-γ), Cytotoxic T-Lymphocyte Associated Protein-4 (CTLA-4), Tumor Microenvironment (TME), Tumor-Associated Macrophages (TAM), Myeloid-Derived Suppressor Cells (MDSC), Regulatory T cells (Treg), Programmed Cell Death Protein-1 (PD-1), Programmed Cell Death Protein Ligand 1 (PD-L1), Antibody Programmed Cell Death Protein-1 (Anti-PD-1), Antibody Programmed Cell Death Protein Ligand 1 (Anti-PD-L1), Phosphatidylinositol-3-kinase (PI3K), Mitogen-Activated Protein Kinase (MAPK), C-C Motif Chemokine Ligand 2 (CCL2), Vascular Endothelial Growth Factor (VEGF), IInterleukin 8 (IL-8), Tumor Mutational Burden (TMB), Innate Anti-PD-1 Resistance Signature (IPRES), Adenosine Monophosphate (AMP), Transforming Growth Factor Gamma (TGF-β), Janus Kinase (JAK).

There are intrinsic mechanisms (Figure 3) in the tumor that contribute to its unresponsiveness to immunotherapy, which can also be developed over the course of the treatment [85,86,87]. The variations in gene expression and signaling pathways in tumor cells and antitumor immune response pathways, that lead to an inhibitory immunosuppressive microenvironment, are some examples of these mechanisms, as described in Figure 3 [86].

The tumors with low levels of TMB, consequently, can present low expression of neoantigens, which can confer resistance to immunotherapy [87,88].

In some tumors, the expression or repression of determined genes and pathways can cause the reduction or loss of antigens expression, preventing immune cell infiltration or function, within the TME and consequently inducing resistance to immunotherapy [85,86]. These mechanisms can be responsible for the acquired resistance by the tumor, by an evolutionary process, selectively reduce the expression of tumor-specific antigens [87]. Also, tumors cells can contribute for the escape to the immunotherapy by changing TME enzymatic activity and metabolism [86,87].

Mitogen-activated protein kinase (MAPK) signalling induces the expression of VEGF and a variety of inhibitory cytokines, such as interleukin (IL)-8, among many other secreted proteins that inhibit T cell function and recruitment [85,86]. This pathway is important in BC pathophysiology, mainly in TNBC development [89]. A study from Loi S et al. with TNBC demonstrated that MAPK activity can suppress the expression of MHC-I and MHC-II, intrinsically and those induced by interferon gamma (IFN-γ), which raises the hypothesis that the activation of MAPK via tumor cells can contribute to circumventing the antigen presentation pathways (Figure 3) [90].

The PI3K/AKT/mammalian target of rapamycin (mTOR) pathways are frequently responsible for uncontrolled tumor cell growth and drug resistance in BC [3]. The tumor suppressor protein phosphatase and tensin homolog (PTEN) is a negative regulator of PI3K signalling and their loss enhances PI3K signalling [3,89]. Also, in these pathways, one of the most frequently mutated genes in BC is the *PI3KCA* gene, which encodes the α isoform of p110 subunit of PI3K [3]. In a murine study, the loss of PTEN was associated with a resistance to PD-1 blockade therapy, conferred by the release of anti-inflammatory cytokines, such as C-C Motif Chemokine Ligand 2 (CCL2) and VEGF, reduced infiltration of CTL cells in tumors, decreased IFN-γ and granzyme B expression (Figure 3). Tumor cells with PTEN mutations tend to be less immunogenic [20,85].

Mutations on *PTEN* and *PI3KCA* gene also contribute to the resistance of tumor cells of mAbs Trastuzumab, Lapatinib and Pertuzumab in HER2+ BC, causing the relapse or metastases of the tumor [3,4,18].

The WNT signalling is divided into canonical and noncanonical WNT pathways that are aberrantly activated in a variety of tumors, like BC, and act in the tumor cell genesis, proliferation, invasiveness, metastatic and immune microenvironment regulation [85,86,91,92]. Some studies demonstrated that, in melanoma tumors and TNBC, the WNT signalling can control the expression of PD-L1 and CTLA-4, as well as have the capacity to regulate the tumor-immune cycle in all steps [92,93,94]. In an experimental murine study, high levels of β-catenin (canonical pathway) in tumors decrease the presence of CD103+ DC, due to a diminutive expression of chemokine that attracts CD103+ DC (CCL4) that causes the prevention of DC migration into the TME and consequently, no antigen-presenting occurs to T cells, contributing to inhibition of activation and cytotoxic effects of T cells. Also, the murine tumors that don’t express β-catenin responded to immune checkpoint therapy whilst tumors that present β-catenin levels did not respond [95].

IFN-γ is a cytokine produced and secreted by effector T cells and APC that act in the activation of IFN receptor signalling molecule Janus kinase 2 (JAK2) and signal transducers and activators of transcription-1 (STAT1), contributing to immune cell activation and regulation of T cell responses, and can also directly induce tumor cell death. Some mechanisms that lead tumor cells to escape immunotherapy are the downregulation or mutating molecules involved in the IFN-γ signalling pathway by tumor cells, like the loss-of-function of genes encoding for JAK1/2 and changes in STAT1 to escape the influence of IFN-γ [86,87]. The JAK1/2 mutations in tumor cells cause the survival of the tumor cells by resisting the antiproliferative effects of IFN-γ [86]. In some studies, it was demonstrated that patients with melanoma who developed resistance to PD-1 therapy acquired loss-of-function mutations in *JAK1/2* that are associated with resistance to treatment [86,96]. In another study, where patients with melanoma treated with Ipilimumab (CTLA-4 inhibitor) were evaluated, the patients who did not respond to the treatment reveal high rates of mutations in the IFN-γ pathway genes, like *JAK2*, *interferon-gamma receptor 1 and 2 (IFNGR1/2)* and *interferon regulatory factor 1 (IRF1)* [86,97].

Tumor cells can adapt to hypoxia environments or vascular circulation mediated by alterations in energy metabolism, named the tumor metabolic reprogramming or the “Warburg effect” [70,86]. The lactate produced by the tumor cells due to metabolic reprogramming can cause the acidification of the TME, affecting the IFN-γ production, NK activation and the amount of MDSC, causing the decrease of the immune response and contributing to the tumor growth [86,98]. Also, the acidosis induced by the tumors can promote the formation of TAM and the upregulation of CTLA-4 expression (Figure 3) on T cells [86]. The Warburg effect is observed in TNBC, causing the immune escape of tumor during the metastasis [70].

The indoleamine-2,3-dioxygenase (IDO) is the enzyme that catalyzes tryptophan degradation into kynurenine and the depletion of tryptophan in T cells by IDO reduces the T cell proliferation, inhibiting the activity of T-cell against tumor cells, as observed in Figure 3) [86,87]. Also, the kynurenine stimulates the proliferation of Treg cells and, in a study with BC samples, it was demonstrated that the increase of IDO expression was associated with the increase of Treg cells [87,99]. The IDO has been suggested as a mechanism of tumor resistance and, in another study, it was demonstrated that IDO can compromise the activity of anti–CTLA-4, anti-PD-1 and anti-PD-L1. In this study, the mice with B16 melanoma were resistant to CTLA-4 treatment but when the CTLA-4 treatment was combined with IDO inhibitor 1- methyltryptophan (1MT) the tumors showed sensitivity to anti-CTLA-4. In mice with B16 melanoma that do not express IDO, a significant reduction in tumor growth and an improvement of survival has been shown [100].

One of the mechanisms that confer primary resistance to immunotherapy is the expression of a certain group of regulatory genes of different processes, like mesenchymal transformation, angiogenesis, extracellular matrix remodelling and others, that are non-responders to anti-PD-1 therapy, namely the innate anti-PD-1 resistance signature (IPRES) [85,86,101].

Other mechanisms that can cause resistance to immunotherapy are the alteration of the antigen presentation pathways by the tumor, which can inhibit the tumor presentation antigens and can be caused by epigenetic changes associated with downregulation of antigen transporters and the inactivation of class I MHC gene transcription [86,87].

Beta-2-microglobulin (B2M) is a protein essential for the transport of MHC class I to the cell surface. When a mutation occurs in the B2M protein, disrupt the antigen presentation and consequently, the recognition of CTL fails, which can lead to acquired resistance to immunotherapy [85,96,102], as described in Figure 3). A study evaluated the genetic alterations associated with acquired resistance to PD-1 blockade in melanoma patients and it was reported a case of B2M mutations were present in resistant tumor cells and lead to a lack of surface expression of MHC class I [96].

The anti-tumor immune response can be suppressed by the tumor and macrophages through the capacity to often release immune-suppressive cytokines, like the transforming growth factor (TGF)-β, that is essential for the angiogenesis and immunosuppression by stimulating Treg cells and are associated with poor prognosis in many tumors [85]. The adenosine is secreted by the upregulation of CD38 and can also be formed by the CD73’s dephosphorylation of adenosine monophosphate (AMP), and the adenosine and CD73 can inhibit the proliferation and function of T cells and promote tumor metastasis [85,86].

### 5.2. Extrinsic Resistance to Immunotherapy

**Figure 4:**
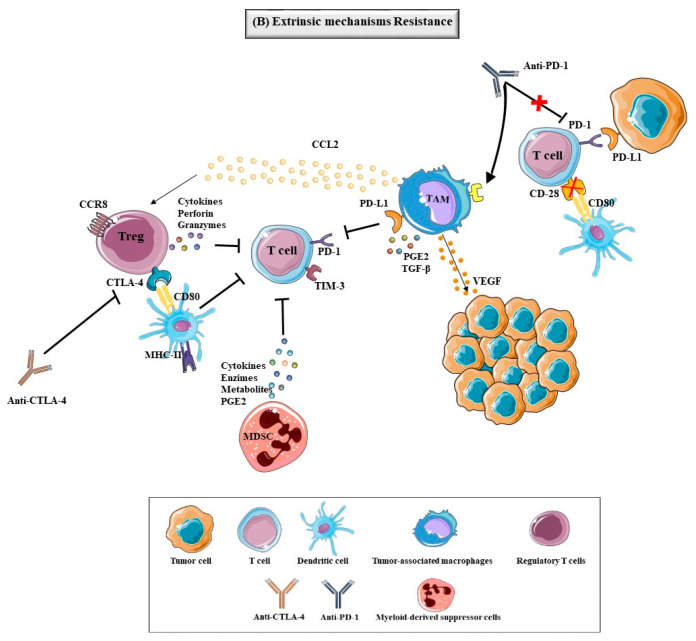
Extrinsic mechanisms of tumor resistance to immunotherapy. The presence of determinate components in TME, like immunosuppressive cells and some molecules, influenced by the tumor can lead to immunotherapy resistance. The Treg cells, characterized by the expression of the FoxP3, are one of these components and they can inhibit MHC molecules and CD80/CD86 on the surface of APC and secrete cytokines, perforin and granzymes that inhibit the activation and proliferation of T cells effectors and APC. The MDSC are a group of cells of type myeloid origin involved in TME, that have a potent immune-suppressive activity by inhibiting the T-cell function with the production of immunosuppressive metabolites, immunosuppressive cytokines, immunoactive enzymes, and immunosuppressive prostaglandin E2 (PGE2), contributing to the progression, invasion and metastasis of the tumor. The TAM (M1 and M2 macrophages) are other types of cells that can infiltrate around tumor cells, due to the action of VEGF and chemokines, and the overexpression of PD-L1, PGE2, TGF-β and CCL2 (promote the accumulation of Treg) affect responses to immunotherapy. In a study, it was demonstrated that TAM can capture anti–PD-1 from the surface of T cells, leading to the resistance of the PD-1 inhibitor. The expression of immune checkpoints can mediate tumor immune resistance, Tim-3 being one of these and expressed in T cells, whereby the upregulation of this immune checkpoint after the PD-1 blockade lead to the inhibition of the activation of T cells, decreasing immunotherapeutic response. The loss of expression of CD28, the homologue of CTLA-4 that promotes the activation of T cells, can cause the T cells lost capacity to proliferate and perform the normal activity, contributing to the immunotherapeutic resistance. Abbreviations are as follows: Cytotoxic T-Lymphocyte Associated Protein-4 (CTLA-4), Antibody Cytotoxic T-Lymphocyte Associated Protein-4 (Anti-CTLA-4), Tumor-Associated Macrophages (TAM), Myeloid-Derived Suppressor Cells (MDSC), Regulatory T cells (Treg), Programmed Cell Death Protein-1 (PD-1), Programmed Cell Death Protein Ligand 1 (PD-L1), Antibody Programmed Cell Death Protein-1 (Anti-PD-1), C-C Motif Chemokine Ligand 2 (CCL2), Vascular Endothelial Growth Factor (VEGF), Transforming Growth Factor Gamma (TGF-β), T cell Immunoglobulin 3 (TIM-3), Prostaglandin E2 (PGE2).

The tumor is capable of inducing resistance to immunotherapy through the extrinsic mechanisms (Figure 4), involving other components of the tumor microenvironment which can affect the immune response, such as inhibitory immune checkpoint, MDSC, M2 TAM and Treg cells [85,86].

The expression of immune checkpoints, such as PD-1, CTLA-4, TIM-3 and V-domain Ig suppressor of T-cell activation (VISTA) in immune cells can mediate tumor immune resistance [85,86]. TIM-3 is a membrane receptor that can be highly expressed in T cells and it was reported, in a study with patients with neck squamous cell carcinoma untreated, that the levels of TIM-3 were upregulated after the PD-1 blockade, causing the inhibiting the activation of T cells and, consequently, a decreasing immunotherapeutic response [102,103]. The increase of TIM-3 on T cells was correlational with the recurrence of the tumor in a mouse model of lung adenocarcinoma, and also in two patients with lung cancer [104,105], Figure 4.

CD28, the homologue of CTLA-4, is a costimulatory receptor that binds to CD80/86 for the activation of the IL-2 and the T cells, promoting the immune responses [41,86,87]. The loss of expression of CD28 can play a role in immunotherapeutic resistance, through the T cells lost capacity to proliferate and perform a normal activity [86,87]. In a study using mice treated with B7 antibody and PD-L1 inhibitor, it was demonstrated that CD28 inhibition contributed to the progression of colon cancer [106], as shown in Figure 4.

MDSC englobe is a group of cells of type myeloid origin, with a morphology similar to neutrophils and monocytic, that express markers, such as CD11b and CD33, but are mostly negative for HLA-DR and have a potent immune-suppressive activity [85,86]. This type of cells has been implicated in TME, contributing to the progression, invasion and metastasis of the tumor by promoting angiogenesis, immune escape and cell growth of the tumor. They can inhibit T-cell function by producing immunosuppressive metabolites, immunosuppressive cytokines, immunoactive enzymes, and immunosuppressive prostaglandin E2 (PGE2) [40,86,107]. The presence of MDSC in TME is correlated with survival decrease in human cancers, such as BC, and the reduced efficacy of immunotherapies, like immune checkpoint therapy [85,108,109]. The γ isoform of PI3K is highly expressed in MDSC cells and induces a transcriptional program that promotes immune suppression during inflammation and tumor growth and contributes to the therapy resistance [85,110,111]. In studies with BC, melanoma and head & neck murine tumor models inactivation of macrophage PI3Kγ in combination with immune checkpoint inhibitors promotes tumor regression and increase survival (Figure 4) [111].

TAM consist of M1 and M2 macrophages, which promote anti-tumor immunity, which have pro-tumorigenic properties, respectively (Figure 3(A3)) [40,85]. These cells infiltrate around tumor cells due to the action of VEGF and chemoIFNkines, and TAM can increase tumor angiogenesis, promote tumor invasion and metastasis [86], as described in Figure 3(A3)). Also, TAM can affect responses to immunotherapy by overexpressing PD-L1, PGE2, and TGF-β and promote the accumulation of Treg by expressing the chemokine CCL22 [40,86]. In a study in vivo, it was demonstrated that TAM can capture anti–PD-1 drugs from the surface of T cells, which leads to the resistance of the PD-1 inhibitor [112].

Treg cells are characterized by the expression of the FoxP3 and, through the inhibition of MHC molecules and CD80/CD86 on the surface of APC, as shown in Figure 3(A3). Maturation of APC and secretion of inhibitory cytokines can inhibit the activation and proliferation of T cells effectors [85,86]. Treg cells can also secrete perforin and granzymes, leading to directly killing T cells and APC. Treg cells are present in many tumors, like BC, where the increase of Treg is correlated to the reduced survival of patients [85,113]. In a study, it was demonstrated that the presence of Treg cells in BC is correlated with higher-grade lesions across all subsets, with high levels of Treg cells in the TNBC in comparison with the others subtypes, and it was suggested that the expression of chemokine receptor CCR8 by Treg cells can be associated with the BC progression [113].

**Figure 5:**
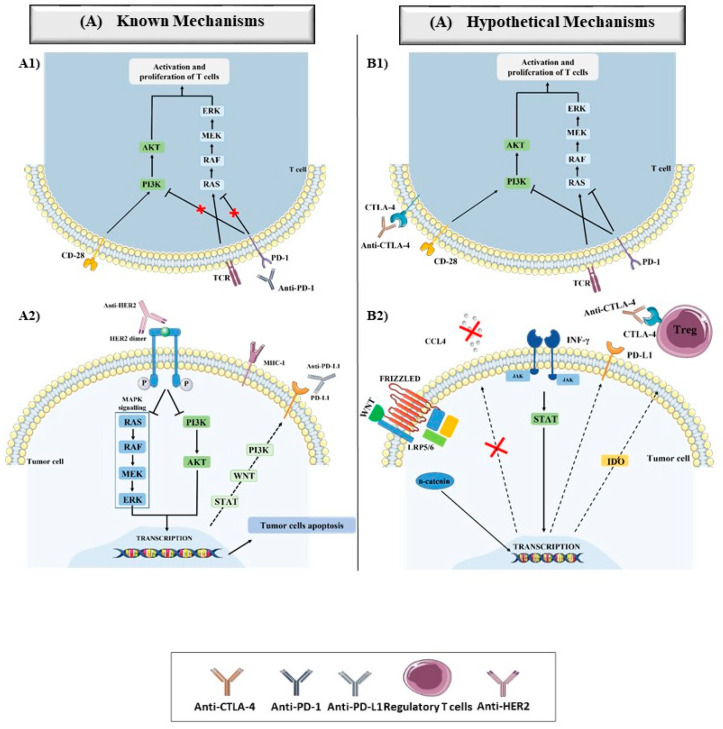
Known and hypothetical target pathways in breast cancer immunotherapeutic treatment. (**A1**) The immune checkpoint PD-1 is one of the mechanisms that can lead to immunotherapy resistance by the downstream of PI3K/AKT and MAPK signaling inhibition, causing the decrease of T cell proliferation and survival, cytokine production, as well as other effector functions. So, when the anti-PD-1 binds to PD-1 expressed in T cells stops the downstream of PI3K/AKT and MAPK signallings inhibition, promoting the activation and proliferation of T cells. Other two mechanisms that promote the activation and proliferation of T cells are the expression of CD28, the homologue of CTLA-4, and the TCR, when they bind to the CD80/86 in APC and MHC-I in tumor cells, respectively, activating the downstream of PI3K/AKT and MAPK signallings; (**A2**) The MAPK and PI3K pathways are the main signaling involved in BC, leading to the proliferation and progression of tumors and antibodies directed for the HER2 protein in tumor cells, inhibiting the activation of the HER2 protein and consequently the activation of the PI3K and MAPK pathways, leading to the apoptosis of tumor cells. Also, the tumor cells can express the immune checkpoint PD-L1 that acts as a mechanism of immunologic escape by the tumor cells when they bind to the PD-1 expressed in T cells. The PD-L1 expression in tumor cells can be stimulated by the INF-γ, WNT and PI3K pathways that can block PD-L1 by the antibodies directed for it, inhibiting the binding with PD-1 in T cells that allows the activation, proliferation and function of T cells against tumor cells; (**B1**) The expression of CTLA-4 by the T cells can limit the T cells activation through the competition with the CD28 for the ligands CD80/86 expressed in APC, and this immune checkpoint can be expressed as a mechanism to escape from host immune surveillance, decreasing the T cells functions. When the anti-CTLA-4 binds to CTLA-4, it allows the APC to bind to the CD28, promoting the activation and proliferation of T cells and immune response. Nowadays, no anti-CTLA-4 is approved as a treatment for BC patients, but some of them are in evaluation for BC patients; (**B2**) Other several mechanisms have been described as a resistance to immunotherapy, like the alterations of some signaling pathways. The alterations in the WNT signalling can lead to the expression of PD-L1 in tumor cells and reduce the expression of chemokine that attracts CD103+ DC (CCL4) by the high levels of β-catenin, decreasing the DC migration into the TME and contributing to the decrease of activation and cytotoxic effects of T cells. The IFN-γ is essential for the immune response when they are secreted by the T cells and APC binds to the receptor in tumor cells, contributing for the immune cell activation and regulation of T cell responses, and can also directly induce tumor cell death. The downregulation or mutations in the molecules involved in the IFN-γ signaling can cause the survival of the tumor cells by resisting the antiproliferative effects of IFN-γ. Tumor cells can also secrete some inhibitory molecules, like IDO enzyme that reduces the T cell proliferation and stimulates the proliferation of Treg cells, contributing to the immunotherapy resistance. The Treg cells also can express the CTLA-4, increasing the immune resistance, and the anti-CTLA-4 can reverse this affect by the mechanisms previously described. Abbreviations are as follows: Cytotoxic T-Lymphocyte Associated Protein-4 (CTLA-4), Antibody Cytotoxic T-Lymphocyte Associated Protein-4 (Anti-CTLA-4), Regulatory T cells (Treg), Programmed Cell Death Protein-1 (PD-1), Programmed Cell Death Protein Ligand 1 (PD-L1), Antibody Programmed Cell Death Protein-1 (Anti-PD-1), Human Epidermal growth Receptor 2 (HER2), Phosphatidylinositol-3-kinase (PI3K), Extracellular regulated kinases (ERK), Mitogen-activated protein kinase (MEK), Protein kinase B (AKT), signal transducers and activators of transcription (STAT), Janus Kinase (JAK), Major Histocompability Complex 1 (MHC-1), T cell Receptor (TCR), Antibody Programmed Cell Death Protein Ligand 1 (Anti-PD-L1), C-C Motif Chemokine Ligand 4 (CCL4), indoleamine-2,3-dioxygenase (IDO).

To overcome resistance to immunotherapy, several approaches have to be taken into consideration. One might be the therapeutic combination with antibodies against PD-1 and CTLA-4 in BC, that in other cancers, such as melanoma, contributed to improved patient survival. This could “rescue” anergized or exhausted T cells to regain functionality and efficacy. On the other side, blocking CTLA-4 can contribute to increase TIL on the BC tumor microenviroment. In Figure 5, we summarize the main known and hypothetical mechanisms that were discussed above, suggesting the combination of different signaling pathways as major targets to overcome breast cancer resistance due to immunotherapy.

## 6. Conclusions

The dynamic interaction between tumor cells and microenvironment associated with tumor heterogeneity led to the complex mechanisms responsible for tumor resistance. Although immunotherapies have been demonstrating clinical benefits in BC, a significant proportion of patients do not respond to the treatment and others initially respond but eventually relapse. Some mechanisms induced by the tumor are responsible for resistance, such as low or the loss of tumor antigens expression, by changing the genes expression, pathways and antigen presentation pathways, the release of immune suppressive cytokines or mold the metabolism for the adaptation to hypoxia environments. Besides these mechanisms, the change of cells in TME contributes to the progression of the tumor after the treatment, compromising the function of immune cells effectors presence, such as Treg cells, MDSC and TAM or the expression or repression of some immune checkpoints in immune cells.

Therefore, resistance is the major challenge for cancer treatment. A strategy for reducing the probability of immune resistance and expanding the effectiveness of immunotherapy is the implementation of a combination of therapies against the tumor. Besides the resistance, the immunotherapy’s success faces other challenges like less immunogenic tumors, the tumor heterogeneity, the complexity of the immune response and the necessity of more biomarkers to predict the clinical benefit of the therapy.

## Figures and Tables

**Table 1:** List of the clinical trials that evaluated monoclonal antibodies, immune checkpoint inhibitors and antibody-drug conjugate in breast cancer patients.

Type	Classification	Treatment	Study Population	Status	Results	Ref.
HER2-directed mAbs						
SOPHIA (Margetuximab) III	HER2 mAbs	Margetuximab + Chemotherapy (QT) vs. Trastuzumab + QT	Metastatic HER2+ BC	Active	PFS: 5.7 months vs. 4.4 months (*p* < 0.001)	[53]
Antibody-Drug Conjugates						
DESTINY-Breast01 (Trastuzumab Deruxtecan)	ADC	Trastuzumab Deruxtecan	Metastatic HER2+ BC received previous treatment with T-DM1	Active	PFSm: 16.4 months (CI 95% 12.7-not reached)	[54]
IMMU-132-01 (Sacituzumab govitecan-hziy)	ADC	Sacituzumab Govitecan-hziy	Advanced epithelial tumors: TNBC	Complete	PFSm: 5.5 months (CI 95% 4.1–6.3)	[55]
NCT02277717 (Trastuzumab Duocarmazine)	ADC	Trastuzumab Duocarmazine	Metastatic solid tumors HER2+: BC	Complete	OR: 33% in HER2+ BC patients (CI 95% 20.4–48.4)	[56]
NCT02222922 (Cofetuzumab Pelidotin)	ADC	Cofetuzumab Pelidotin	Advanced Solid Tumors: TNBC	Complete	ORR: 21% TNBC patients (CI 95% 8–40)	[57]
Immune Checkpoint Inhibitors						
IMpassion130 (Atezolizumab)	Anti-PD-L1	Atezolizumab + Nab-paclitaxel vs. Placebo + Nab-paclitaxel	Untreated Metastatic TNBC	Complete	PFSm: 7.2 months vs. 5.5 months (*p* = 0.002) PFS patients PD-L1 > 1%: 7.5 months vs. 5 months (*p* < 0.001)	[58]
Javelin (Avelumab)	Anti-PD-L1	Avelumab	Metastatic or locally advanced solid tumors: Metastatic BC	Complete	ORR: 3% MBC patients (CI 95% 1.0–6.8) and 5.2% TNBC patients (CI 95% 1.1–14.4)	[59]
KEYNOTE-086 Cohort-A (Pembrolizumab)	Anti-PD-1	Pembrolizumab	Treated Metastatic TNBC	Complete	ORR: 5.3% total TNBC patients (CI 95% 2.7–9.9) and 5.7% TNBC PD-L1+ (CI 95% 2.4–12.2)	[60]
KEYNOTE-086 Cohort-B (Pembrolizumab)	Anti-PD-1	Pembrolizumab	Untreated Metastatic TNBC PD-L1+	Complete	ORR: 21.4% (IC 95% 13.9–31.4)	[61]
KEYNOTE-522 (Pembrolizumab)	Anti-PD-1	Pembrolizumab + QT vs. Placebo + QT	Untreated Early-stage TNBC	Active	pCR: 69.5% (CI 95% 59.9–69.5) vs. 51.2% (CI 95% 44.1–58.3)	[62]
KEYNOTE-355 (Pembrolizumab)	Anti-PD-1	Pembrolizumab + QT vs. Placebo + QT	Untreated Recurrent Inoperable or Metastatic TNBC	Active	PFSm: 9.7 months vs. 5.6 months CPS (PD-L1) ≥ 10 patients (*p* = 0.0012)	[63]
KEYNOTE-028 (Pembrolizumab)	Anti-PD-1	Pembrolizumab	ER+/HER2- metastatic BC PD-L1+	Complete	ORR: 12% (CI 95% 2.5–31.2)	[64]
PANACEA KEYNOTE-014 (Pembrolizumab)	Anti-PD-1	Pembrolizumab	Advanced HER2+ BC	Complete	OR: 15% patients PD-L1+ (CI 90% 7–29)	[65]
ICON (Ipilumumab and Nivolumab)	Anti-CTLA-4 Anti-PD-1	Ipilumumab + Nivolumab + QT	Metastatic HR+ BC	Active	No results	[66]

Abbreviations are as follows: hormone receptors (HR), human epidermal growth receptor 2 (HER2), triple negative breast cancer (TNBC), monoclonal antibodies (mAbs), breast cancer (BC), versus (vs.), cytotoxic T-lymphocyte associated protein-4 (CTLA-4), chemotherapy (QT) programmed cell death protein-1 (PD-1), programmed death-ligand 1 (PD-L1), objective responses (OR), progression-free survival (PFS), median progression-free survival (PFSm), objective response rate (ORR), pathologic complete response (pCR), confidence interval (CI).

## Data Availability

Not applicable.
